# Adverse cardiac events in the treatment of non-small cell lung cancer with programmed death-1and programmed death-ligand 1 inhibitors: A protocol for systematic review and meta-analysis

**DOI:** 10.1097/MD.0000000000021613

**Published:** 2020-08-07

**Authors:** Honglin Li, Deting Han, Xiaoteng Feng, Wenjun Yu, Tongtong Xu, Tao Ma, Lucheng Song

**Affiliations:** a First Clinical College; b College of Traditional Chinese Medicine, Shandong University of Traditional Chinese Medicine; c The First Affiliated Hospital of Shandong First Medicial University (Shandong Provincial Qianfoshan Hosipital, Shandong University), Jinan, Shandong, China.

**Keywords:** adverse cardiac events, meta-analysis, non-small cell lung cancer, programmed death-1, programmed death-ligand 1, protocol, systematic review

## Abstract

**Background:**

Programmed death-1 (PD-1) and programmed death ligand-1 (PD-L1) inhibitors are immune therapies that have shown great promise in the treatment of multiple cancers. However, immune-related adverse events of PD-1 and PD-L1 inhibitors may limit their use in non-small cell lung cancer (NSCLC). Given the rising number of clinical trials in recent years, it is essential to perform a meta-analysis to provide assess the cardiotoxicity of PD-1/ PD-L1 inhibitors in NSCLC therapy.

**Method and analysis:**

The ClinicalTrials.gov, Embase, PubMed, and Cochrane Central Register of Controlled Trials repositories will be searched from their inception to December 2019. The bibliography of the searching process will be imported into Endnote X9 software. Two reviewers independently will screen the literature, extract data, and conduct the risk of bias for every added study. The data analysis will be analyzed using Stata15.0 software. Specific adverse cardiac events will be identified, with particular attention on atrial fibrillation, cardiac arrest, cardiac failure, and pericarditis. This review will be performed as per the Preferred Reporting Item for Systematic Review and meta-analysis statement recommendations.

**Ethics and dissemination:**

This study will provide support for the cardiotoxicity linked to the treatment of NSCLC using PD-1/PD-L1 inhibitors. The data in the meta-analysis will be retrieved from completed and published clinical trials; therefore, ethical review and patient informed consent will not be required.

**PROSPERO number:**

CRD42020156397.

## 1 Introduction

Lung cancer (LC) constitutes the major genesis of cancer-associated globally^[1]^. In terms of pathological types, most LCs are non-small cell LC (NSCLC), responsible for an estimated 85%^[2]^. The World Health Organization has classified NSCLC into 3 major classes, including adenocarcinoma, squamous cell carcinoma, and large cell.^[3–5]^ Stages of the disease determine the treatment options for NSCLC.^[6]^ Therefore, stage I-II NSCLC is primarily treated surgically, and advanced NSCLC is treated using platinum-containing doublet chemotherapy as the first line of treatment.^[7]^ Nevertheless, resistance to platinum-based chemotherapy is an essential factor affecting the therapeutic effect.^[8]^

With the advent of Immune Checkpoint Inhibitors (ICIs), specially programmed death-1 (PD-1) inhibitors (Pembrolizumab, Nivolumab), and programmed death-ligand 1 (PD-L1) inhibitors (Durvalumab, Avelumab, and atezolizumab), the therapeutic landscape of immunotherapy has been changed in NSCLC.^[9]^ PD-1/PD-L1 inhibitors have revealed a remarkable impact in overall survival relative to chemotherapy in NSCLC.^[10,11]^

PD-1 is expressed in diverse immunity cells, a modulator of T cells, consisting of T cells and B cells.^[12]^ PD-L1 and PD-L2, the ligands of PD-1, are expressed in the immune cells, as well as in the tumor cells^[13]^. The immune system and Treg up-regulation are inhibited when PD-1 binds to PD-L1/PD-L2 in the tumor microenvironment; hence, the immune surveillance system cannot fully play its role in eliminating malignant tumor cells.^[14]^ Immune evasion occurs when tumor cells overexpress PD-L1.^[15,16]^ ICIs repress the binding of PD-1 as well as PD-L1, and reactivate cytotoxic T cells to kill cancer cells.^[17]^ However, the immune balance is disrupted when the immune system is activated, and normal tissues are over-attacked. Therefore, recent studies have focused on immune-related adverse events, including cutaneous, pneumonitis, gastrointestinal, and endocrine effects.^[18]^ The findings of a study in mice indicated that the inhibition of the PD-1 leads to dilated cardiomyopathy.^[19]^ Research evidence shows that ICI is associated with cardiotoxicity of NSCLC, including myocarditis, cardiac conduction abnormalities, and pericardial tamponade.^[20–22]^ PD-1, as well as PD-L1, are synthesized in human cardiomyocytes, and PD-L1 expression was detected in damaged cardiomyocytes of patients with adverse cardiac events.^[23]^ Most adverse cardiac events are managed using high doses of glucocorticoids.^[24]^ Unlike other adverse events, cardiotoxicity is life-threatening. Therefore, the tradeoff between safety and efficacy and the potential risk of toxicity to the heart should be considered. In the most recent meta-analysis, the findings showed no marked differences in cardiotoxicity between immunotherapy and chemotherapy.^[25]^ This is because individual adverse cardiac events were not analyzed, and only 3 clinical trials were included with the retrieval date set for February 2017. In recent years, several clinical trials have been conducted and results published in peer-reviewed journals.

This meta-analysis will address the cardiac-related adverse events induced by PD-1/PD-L1 inhibitors, and compare PD-1/PD-L1 inhibitors with chemotherapy. Here, more clinical randomized controlled trials will be added, and individual cardiac-related adverse events will be detailed.

## 2 Methods

### 2.1 Study indexing

This meta-analysis method has been indexed on Prospero (CRD42020156397). We will perform this review as per the Preferred Reporting Item for Systematic Review and meta-analysis statement guidelines.^[26]^^.^

### 2.2 Criteria for including randomized controlled trails (RCTs)

#### 2.2.1 Types of studies

This review will consist of all randomized RCTs using PD-1 or PD-L1 inhibitors for NCLC patients, without the restriction of publication type, and the language will be limited to English. Reviews, animal experiments, descriptive studies, case reports, Non-RCTs, quasi-RCTs, and uncontrolled trials will not be added.

#### 2.2.2 Subjects

All the patients diagnosed with NCLC and treated with PD-1/PD-L1 inhibitors. The types and stages of NSCLC will not be restricted.

#### 2.2.3 Types of interventions

Experimental interventions: The intervention will consist of PD-1/PD-L1 inhibitors, combined with chemotherapy or not. PD-1/PD-L1 inhibitors, combined with cytotoxic T-cell antigen-4 suppressors, such as Ipilimumab, will be excluded.

Control interventions: The control interventions will include chemotherapy. However, other immuno-checkpoint inhibitors, cytotoxic T-cell antigen-4 inhibitors, will be excluded.

#### 2.2.4 Types of outcome parameters

The clinical endpoints will constitute incidence and relative risk of specific cardiovascular adverse events of PD-1/PD-L1 inhibitors versus chemotherapy in non-small cell LC, including atrial fibrillation, cardiac arrest, cardiac failure, pericarditis, and pericardial tamponade.

### 2.3 Search methods for identifying RCTs

#### 2.3.1 Search strategy

We will systematically search for RCTs in 3 English medical databases: Cochrane Central Register of Controlled Trials, PubMed, and Embase from the inception period to September 2019. To avoid missing any eligible RCTs, the grey literature in ClinicalTrials.gov and reference lists of identified articles will be searched based on snowball approach. The search approach of PubMed is shown in Table 1.

**Table 1:**
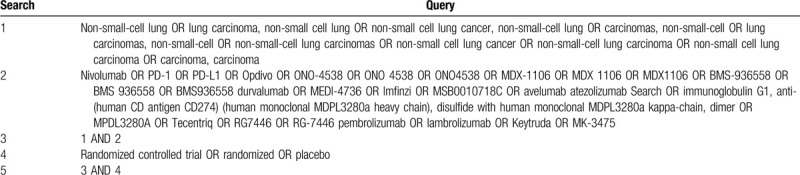
Search strategy in PubMed database.

#### 2.3.2 Study selection

All bibliography from the searching process will be imported into Endnote X9 software. Two reviewers (HLL and DTH) will independently screen the literature. The literature will be selected according to the inclusion criteria. If there are discrepancies, the 2 reviewers will discuss them with a third reviewer (LCS) to resolve. First, we will screen each title and abstract of all studies. Second, we will investigate the full text to exclude irrelevant literature. If necessary, contacting the original study authors by Email or phone to obtain unidentified information will be important for this study.

#### 2.3.3 Data collection and management

The 2 reviewers (HLL and DTH) will complete the data extraction independently according to a predefined data extraction template. If there will be any disagreement, we will resolve it with the third reviewer (LCS) through discussion. We will add the following data from individual RCTs on the data retrieval forms:

(1) Study characteristics: title, first author, journal, year of publication, country, fund support, and so on.
(2) Methodology information: study design, assignment blinding, concealment, incomplete outcome data, selective reporting, sample size computation, baseline relativity, and so on.
(3) Participant population: age, gender, ethnicity, type of surgical procedure, type of tumor, disease duration, comorbidity, inclusion criteria, exclusion criteria, and so on.
(4) Intervention information: doses, regimen, scheme, duration, and so on.
(5) Outcome measures: cardiac-related adverse events, all-cause mortality, and so on.

### 2.4 Evaluation of risk of bias in added RCTs

The risk of bias of each RCT added will be conducted singly by 2 reviewers (HLL and DTH) based on tool of Cochrane collaboration for examining the risk of bias. We will use the following items in assessing the bias in each trial: selective outcome reporting (reporting bias); random sequence determination (selection bias); assignment seclusion (selection bias); incomplete outcome data (attrition bias); concealing of patients and personnel (performance bias); concealing of outcome inspection (detection bias); other bias.^[27]^ Above 7 aspects will be reported as “low risk, uncertain risk, high risk”.

### 2.5 Data analysis

The data analysis will be analyzed using Stata 15.0 software. Different effect indicators will be selected according to different types of research design and data. Binary data will be analyzed using the odds ratio or relative risk with 95% confidence intervals (CI), continuous data will be shown using mean difference or standard mean difference with 95% CI. I square (*I*^2^) statistic will be used to evaluate the diversity of the literature. When I^2^ <50%, the fixed-effects model will be used, otherwise (*I*^2^> 50%), the random-effects model will be adopted. If the outcome indicators were included in 10 or more than 10 trials, the Egger test will be used to inspect whether publication bias existed or not. Symmetries mean that publication bias is less likely; otherwise, we will explain the reason for asymmetries.

### 2.6 Subgroup examination

Subgroup evaluation aims to identify sources of heterogeneity and to analyze the data further. In case of high heterogeneity, subgroup analysis will be performed based on distinct characteristics of included RCTs.

### 2.7 Sensitivity analysis

Sensitivity analysis checks the stability of the results by selecting another research indicator or changing a different analysis model. If the statistical results do not change significantly with the statistical models and indicators, the results are relatively stable and credible.

### 2.8 GRADE quality assessment

We will evaluate the quality of evidence using GRADE Pro V.3 software according to GRADE handbook.^[28]^ The evaluation will be examined from limitations, inconsistency, indirectness, imprecision, as well as publication bias. The results of evidence will be assigned into 4 grades, that is, very low quality, low quality, moderate quality, and high quality.

## 3 Discussion

NSCLC is more insensitive to chemotherapy than small cell LC (SCLC), then chemotherapy and targeted treatments tend to acquire resistance. ICIs, particularly PD-1/PD-L1 inhibitors, are effective in treating melanoma,^[29]^ renal cell carcinoma,^[30]^ and colorectal cancer.^[31]^ Research evidence shows that PD-1/PD-L1 blockades have improved overall survival (OS) than docetaxel for NSCLC^[32]^. The mechanism of PD-1 and PD-L1 inhibitors involves mainly restarting the T cell-mediated tumor cell death process; at the same time, interferes with the normal immune system. In a meta-analysis, the authors divided patients into NSCLC group and other cancer groups (melanoma, prostate cancer), that is, based on the cancer types and indicated that PD-1/PD-L1 inhibitors have no significant differences in cardiotoxicity compared with chemotherapy or placebo^[25]^. However, since the search date was limited to February 2017, only 3 studies of NSCLC were included. Several clinical RCTs results have been conducted over the last 2 years, and there have been several cases of adverse cardiac events in the treatment of NSCLC. Although cardiotoxicity is an uncommon immune-related adverse events, it could have an impact on patient survival. Therefore, a meta-analysis involving more studies is required to provide evidence for the cardiotoxicity of PD-1/PD-L1 suppressors.

## Author contributions

HLL and DTH contributed equally as co-first authors.

**Conceptualization:** Honglin Li, Deting Han.

**Data curation:** Honglin Li, Deting Han, Xiaoteng Feng, Lucheng Song.

**Formal analysis:** Honglin Li, Deting Han.

**Funding acquisition:** Honglin Li, Deting Han, Lucheng Song.

**Investigation:** Honglin Li, Wenjun Yu, Tongtong Xu, Tao Ma.

**Methodology:** Honglin Li, Deting Han.

**Software:** Honglin Li, Deting Han.

**Supervision:** Honglin Li, Deting Han, Lucheng Song.

**Validation:** Honglin Li, Deting Han, Xiaoteng Feng, Wenjun Yu, Tongtong Xu, Tao Ma, Lucheng Song.

**Visualization:** Honglin Li, Deting Han.

**Writing – original draft:** Honglin Li, Deting Han.

**Writing – review & editing:** Honglin Li, Deting Han, Lucheng Song.
